# Biochemical and aggregation analysis of Bence Jones proteins from different light chain diseases

**DOI:** 10.1080/13506120701815324

**Published:** 2008-02-22

**Authors:** Laura A. Sikkink, Marina Ramirez-Alvarado

**Affiliations:** Department of Biochemistry and Molecular Biology, Mayo Clinic College of Medicine, Rochester, Minnesota, USA

**Keywords:** Light chain amyloidosis, light chain deposition disease, multiple myeloma, circular dichroism, electron microscopy, fibrils, aggregates, amyloid

## Abstract

Deposition of immunoglobulin light chains is a result of clonal proliferation of monoclonal plasma cells that secrete free immunoglobulin light chains, also called Bence Jones proteins (Bence Jones proteins). These Bence Jones proteins are present in circulation in large amounts and excreted in urine in various light chain diseases such as light chain amyloidosis (AL), light chain deposition disease (LCDD) and multiple myeloma (MM). BJP from patients with AL, LCDD and MM were purified from their urine and studies were performed to determine their secondary structure, thermodynamic stability and aggregate formation kinetics. Our results show that LCDD and MM proteins have the lowest free energy of folding while all proteins show similar melting temperatures. Incubation of the BJP at their melting temperature produced morphologically different aggregates: amyloid fibrils from the AL proteins, amorphous aggregates from the LCDD proteins and large spherical species from the MM proteins. The aggregates formed under in vitro conditions suggested that the various proteins derived from patients with different light chain diseases might follow different aggregation pathways.

## Introduction

Light chain amyloidosis (AL), light chain deposition disease (LCDD) and multiple myeloma (MM) are immunoproliferative disorders characterized by excessive proliferation of monoclonal plasma cells, excretion of high amounts of Bence Jones proteins (BJP) and deposition of immunoglobulin light chains. BJP are soluble free monoclonal immunoglobulin light chains that are secreted into circulation and excreted in urine [1]. The symptoms and tissue damage vary between these three light chain diseases. AL is characterized by the deposition of immunoglobulin light chains as amyloid fibrils in vital organs leading to organ failure. The organs most commonly affected include the kidney, heart and liver [2]. AL can also affect tissues such as peripheral nerve, gastrointestinal tract and pulmonary [3]. LCDD is characterized by granular amorphous aggregates in the basement membrane of the kidney. The kidney is the most frequently affected organ, but LCDD can also occur in other organs such as the heart and liver, where it is usually asymptomatic [4]. MM is characterized by bone lesions, hypercalcemia, renal failure and anemia. MM can form casts in the kidney that can lead to renal complications [5]. Lambda light chains are more prevalent in AL, while kappa light chains are more prevalent in LCDD and MM [6,7].

The ability of BJP to aggregate could be caused by numerous factors, in particular the combination of thermodynamic instability due to somatic mutations, germline context and/or proteolysis [8]. According to Hurle et al., domain stability of proteins plays an important role in fibrillogenesis. The less stable domains tend to form aggregates and any mutation that decreases domain stability could lead to AL [9].

There is a bias for certain germline sequences towards amyloidogenicity. AL has a preference for certain germline sequences including VλIII 3r, VλVI 6a, VκI O18/O8 and VκIV B3 [6]. In LCDD, there is a preference for VκIV B3 [10]. Some possible explanations for this bias include the fact that certain germline gene families are more available, leading to recombination events and clonal expansion [6]. It is also possible that some germline genes are selected due to their antigen diversity.

The majority of AL amyloid fibrils are made up of N-terminal fragments corresponding to the variable domain, suggesting the possibility of a proteolytic event. Partial proteolysis could destabilize proteins and predispose them to form aggregates [8].

In this study, we compared the protein structure, protein stability and aggregation properties of light chain proteins from AL, LCDD and MM patients in order to gain an understanding about the similarities and differences between BJP involved in these different light chain deposition diseases.

## Methods

### Cloning

Bone marrow cells were collected following the guidelines from the Institutional Review Board at the Mayo Clinic. RNA extracted from patients' bone marrow cells was used in a reverse transcriptase polymerase chain reaction (RT-PCR) to produce cDNA. The cDNA was amplified with degenerate primers encoding the N-terminus of the germline sequences by PCR (six different primers for lambda proteins and three different primers for kappa proteins) as previously reported [6]. Light chain sequences were analyzed using Vbase (http://www.mrccpe.cam.ac.uk) to determine the germline donor sequence for each DNA variable domain sequence. Specific germline primers corresponding to the variable light chain gene were used to amplify the gene corresponding to the light chain variable domain by PCR and then cloned into pCR®II TOPO® vector following the protocol from the TOPO TA cloning®kit (Invitrogen, Carlsbad, CA, USA). The DNA Synthesis and Sequencing Core Facility at the Mayo Clinic synthesized primers and sequenced plasmids. DNA sequences were verified in Vbase and by BLAST 2 sequence alignment (www.ncbi.nlm.nih.gov/blast/bl2seq/wblast2.cgi) and the sequences were translated into their amino acid sequence using ExPasy (www.us.expasy.org/tools/dna.html) and mutations in the patient sequence were noted in comparison to the germline. The sequences have been deposited in GenBank with the following accession numbers DQ240234 (AL-02), DQ240235 (AL-03), DQ240236 (MM-01), DQ240237 (MM-02), DQ240238 (LCDD-01), and DQ240239 (LCDD-02, predominant VκI O12/O2 sequence).

### Protein purification

Patients' urine samples were collected following the guidelines from the Institutional Review Board at the Mayo Clinic. Patients' urine samples were dialyzed in nanopure water overnight at 4°C. The dialyzed urine was filtered with 0.22μmm disposable filter and 0.02% sodium azide was added. BJP were purified by size exclusion chromatography using a HiLoad 16/60 Superdex 75 prep grade column on an AKTA FPLC (GE Healthcare, Piscataway, NJ, USA) in 10 mM Tris–HCl pH 7.4 buffer. Pure fractions were checked by SDS polyacrylamide gel electrophoresis (SDS-PAGE) and stained with Coomassie blue. The extinction coefficient for each protein was calculated using the biopolymer calculator (http://paris.chem.yale.edu/cgi-bin/extinct.pl) which was used with its absorbance at 280 nm to determine the protein concentrations. Pure fractions were combined, concentrated and stored at 4°C.

### Molecular weight determination

The molecular weight of the purified protein was determined on a Superdex 75 10/30 size exclusion column on an AKTA FPLC (GE Healthcare) with 10 mM Tris–HCl pH 7.4 and 150 mM NaCl buffer. Molecular weight standards of albumin, carbonic anhydrase, cytochrome C and aprotinin (Sigma, St Louis, MO, USA) were prepared in 10 mM Tris–HCl pH 7.4 and 150 mM NaCl buffer with a concentration of 3 mg/ml for aprotinin and carbonic anhydrase and a concentration of 2 mg/ml for albumin and cytochrome C. The column was calibrated with each standard at a flow rate of 0.3 ml/min. The elution volume for each standard peak was determined. The purified BJP were injected and eluted through the column at a flow rate of 0.3 ml/min. Blue dextran, concentration of 1 mg/ml, was injected onto the column to determine its void volume, which is used to calculate the ratio of elution to void volume (Ve/Vo) for each molecular weight standard. A calibration curve was produced by plotting the logarithms of each standard as a function of their Ve/Vo and determining the line of best fit. After determining the Ve/Vo of each BJP, the molecular weight was calculated by using the equation for the line of best fit.

### Circular dichroism spectroscopy

Protein secondary structure was measured by Far UV-CD spectra (260–200 nm) on an AVIV 215 circular dichroism (CD) spectrometer (AVIV Biomedicals Inc., Summerset, NJ, USA) in the continuous mode by taking measurements every 1 nm with an averaging time of 5 s at 4°C in a 0.2 cm path-length cuvette. Protein concentrations varied between 5.5–25.6 μM. Thermal denaturations were carried out following the ellipticity at 218 nm (maximum β-sheet signal) for each protein. The ellipticity was monitored at 2°C intervals from 4 to 90°C with an equilibration time of 1 min and an averaging time of 30 s in a 0.2 cm path-length cuvette. A refolding curve was monitored immediately after the unfolding curve acquisition from 90 to 4°C using the same parameters as previously stated. Thermal denaturation data were processed following a two-state transition model. Folded and unfolded baselines were linear extrapolated from the data with a minimum of 10 points for the majority of the proteins. In some cases (uMM-02), we used fewer points for the unfolded baseline. The fraction folded (FF) at each temperature was calculated by using the following equation:

$$\begin{array}{lll} \text{FF} & = & (\text{ellipticity observed}-\text{ellipticity of the unfolded})/\\ & & (\text{ellipticity of folded}-\text{ellipticity of unfolded}). \end{array}$$

Melting temperature (T_m_) was calculated at the midpoint transition for each protein. ΔG was calculated according to the equation

$$\begin{array}{lll} \Delta \text{G}(\text{T}) & = & \Delta {\text{H}}_{\text{m}}-\left(1-{\text{T}/\text{T}}_{\text{m}}\right)\\ & & -\Delta {\text{C}}_{\text{p}}\left[\left({\text{T}}_{\text{m}}-\text{T}\right)+\text{T}\;\text{ln}\;\left({\text{T}/\text{T}}_{\text{m}}\right)\right] \end{array}$$

from Pace where ΔH is derived from the van't Hoff equation:

ln Keq=(-ΔH/R)*(1/T)+(ΔS/R) and  ΔCp≈172+17.6*N-164*SS  (in cal mol−1K-1)

where N is the number of amino acid residues and SS is the number of disulfide cross-links in the protein [11]. An alternative method tested used the points in the FF transition to determine the free energy of folding. The equilibrium constant (Keq) was calculated from these points. ΔG_folding_ was calculated using the following equation:

$$\Delta {\text{G}}_{\text{folding}}=-\text{RT}\;\text{ln}\;\text{Keq}$$

where R = gas constant (1.98 cal mol^−1^ K^−1^), T = temperature in Kelvin. ΔG(4°C) was calculated by extrapolating free energy of folding versus the temperature to determine the line of best fit for the free energy of folding at 4°C/277 K. Both methods gave rise to very similar ΔG_folding_ values.

Chemical denaturations were done at 4°C for all proteins except for uAL-03, which was done at 22°C in a 0.2 cm path-length cuvette. A Far UV-CD spectra of the folded (absence of urea) and unfolded (presence of urea) samples were collected to determine the wavelength with the maximum ellipticity difference between them. Ellipticity at that wavelength (218 nm) was monitored in 1 s intervals during a 60 s scan, with an equilibration time of 10 min prior to the scan. The data were averaged for each urea concentration. Denaturation curves were obtained by mixing equal volumes of two protein stock solutions, in the presence and absence of urea, to change the urea concentration while keeping the protein concentration constant. Both the initial and final urea concentrations were confirmed by refractometry and calculated using the equation

$$\begin{array}{lll} \left[\text{urea}\right] & = & 117.66*\left(\Delta \text{N}\right)+29.753*{\left(\Delta \text{N}\right)}^{2}\\ & & +185.56*{\left(\Delta \text{N}\right)}^{3}, \end{array}$$

where ΔN is the difference in refraction between the sample and buffer [11]. The denaturation curves were processed the same way as the two-state transition model used in the thermal denaturation to calculate fraction folded curves. The melting concentration of denaturant (C_m_) was determined at the midpoint transition of the unfolding curve. ΔG_folding_ was calculated using the same equation as used with the thermal denaturation data. Free energy of folding versus the concentration of urea was extrapolated to determine the line of best fit for the free energy of folding in the absence of denaturant (ΔG(H_2_O)).

### ANS binding assay

A stock solution of ANS (8-anilino-1-napthalene-sulfonic acid) was prepared, filtered and its concentration determined by checking its absorbance at 350 nm and using an extinction coefficient of 5000 (M * cm)^−1^ [12]. A 5 μM protein sample was prepared with 2.3 μM ANS in 10 mM Tris–HCl pH 7.4 buffer in the presence and absence of 6 M urea. Samples were placed in a quartz cuvette with a path-length of 1 cm in a temperature-controlled Model QM-2001 fluorometer (Photon Technology International, Lawrenceville, NJ, USA). ANS fluorescence was monitored at an excitation wavelength of 370 nm and by an emission wavelength scan from 400–620 nm with slit widths of 7 and 8 nm at a temperature of 26°C.

### Western blot

Urine samples, pure BJP and known positive controls were run on a SDS-PAGE gel and transferred to an Immobilon P (PVDF) membrane (Millipore, Billerica, MA, USA) using a semidry transfer apparatus for 1 h. The membrane was blocked for 1 h in PBS with 5% non-fat dry milk rocking at room temperature. The membrane was rocked overnight at 4°C with 1:500 dilution of the primary antibody (anti-human kappa (AFF) or anti-human lambda (AFF); The Binding Site, San Diego CA, USA) in blocking buffer. The membrane was washed with two washes of PBS, three washes of PBS + 0.1% Tween 20 followed by one wash with PBS. It was rocked for 1 h with a 1:8000 dilution of secondary antibody conjugated to horseradish peroxidase (HRP; rabbit polyclonal to sheep IgG H + L (HRP); Novus Biologicals, Littleton, Co, USA) in blocking buffer at room temperature. The membrane was washed again with PBS and PBS + 0.1% Tween 20 following the previous wash method listed above. Antibody bound to the protein was detected using an ECL chemiluminescence reagent (GE Healthcare) and exposed to film.

### Aggregate formation

Aggregate formation assays incubated at the T_m_ of each protein were set up with 5 μM protein in 10 mM Tris–HCl pH 7.4 buffer with 15 μM thioflavine T (ThT) with a final volume of 1.5 ml. Samples were placed in a quartz cuvette with a stir bar and incubated at their T_m_ (57°C for uAL-02, uAL-03, uMM-01 and uMM-02, 60°C for uLCDD-01 and uLCDD-02) in a temperature-controlled Model QM-2001 fluorometer (Photon Technology International). Tryptophan fluorescence was monitored using an excitation wavelength of 294 nm and emission wavelength of 350 nm. ThT fluorescence was monitored using an excitation wavelength of 450 nm and emission wavelength of 480 nm. The two different probes were followed simultaneously for 72 h with continuous stirring at 300 rpm. Shutters were open only during the acquisition. All reactions were measured in a 1 cm path-length cuvette with slit widths of 3 nm, taking an acquisition every 25 s with an integration time of 0.5 s.

Aggregate formation assays incubated at 37°C were set up with 5 μM protein in 10 mM Tris–HCl pH 7.4 with 5 μM ThT in triplicate in a 384-well, flat-bottomed, high-binding, polystyrene Costar plate (Corning Incorporated, Corning, NY, USA) in a GENios FL plate reader (TECAN US, Durham, NC, USA). The plate was incubated at 37°C with a reading occurring every 15 min with 3 min of shaking prior to each reading.

### Electron microscopy (EM)

Endpoint samples from the aggregation experiments were centrifuged and resuspended in 100 μl of buffer. Three microliters of the concentrated aggregate was placed on a 300-mesh copper formvar/carbon grid and negatively stained with either 4% uranyl acetate or 1% phosphotungstic acid and examined on a JEOL 1200 EX transmission electron microscope.

### Amino acid analysis

Ten micrograms of each protein were sent to Scientific Research Consortium, Inc. in St. Paul, Minnesota where acid hydrolysis was performed.

### Trypsin digestion/tandem mass spectrometry and mass spectrometry intact mass analysis

Twenty-five micrograms of each protein were taken to the Mayo Clinic Proteomics Research Center to carry out trypsin digestion/tandem mass spectrometry and intact mass analysis. The proteins to be analyzed by nanoLC-ESI-tandem mass spectrometry were initially reduced and alkylated with DTT and iodoacetamide, digested with trypsin and the peptides run on a ThermoFinnigan LTQ Orbitrap. The MS/MS spectra were searched with Sequest using both Swiss-Prot database and the expected peptides from the sample protein sequences. Intact mass measurements were made by LC-MS using a C18 reverse-phase HPLC column eluting into an Agilent LC/MSD TOF mass spectrometer.

## Results

**Figure 1:**
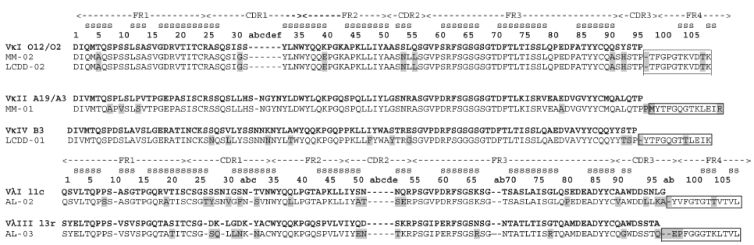
Sequence alignment of the variable and junction domain portions of the BJP. Donor germline sequence for each Bence Jones protein (BJP) was determined by PCR and sequence analysis. DNA sequences were translated into the amino acid sequence. Mutations in each protein sequence when compared to the germline sequence are highlighted in gray. The junction domain is highlighted with a box. FR, framework region; CDR, complementarity determining region; s, β-strand. The constant domains for AL and MM proteins are not shown here. Kappa proteins (MM) have only one constant domain. The constant domain for AL-02 is IGLC1 and for AL-03 it is IGLC2.

We studied two BJP for each type of light chain disease. These BJP were called uAL-02, uAL-03, uLCDD-01, uLCDD-02, uMM-01 and uMM-02. The ‘u’ denotes these proteins were derived from a patient's urine sample and the name without the ‘u’ denotes the corresponding DNA sequence name. Table I contains information for each protein studied such as the germline sequence, disease, organ involvement, year of diagnosis and confirmation of diagnosis for each protein. Each protein sequence was translated from the DNA sequence and compared with its corresponding germline to note any mutations which are highlighted in gray in Figure 1. AL-02 has a total of 19 mutations, AL-03 has 17 mutations, LCDD-01 has 11 mutations, LCDD-02 (VκI O12/O2) has nine mutations, LCDD-02 (VκI L1) has eight mutations, MM-01 has seven mutations and MM-02 has nine mutations.

**Table I:** Protein comparison table.

Protein	Isotype	Germline	Disease	Diagnosis year	Diagnosis confirmed	Organ(s)/tissue involved	Molecular weight (κDa)
uAL-02	Lambda	λI-1c	AL	2003	Endomyocardial biopsy	Heart, liver	25
uAL-03	Lambda	λIII-3r	AL	2003	Renal biopsy	Renal	25
uLCDD-01	Kappa	κIV-B3	LCDD	2004	Renal biopsy	Renal	12
ULCDD-02	Kappa	κI-012/02	LCDD	2004	Renal biopsy	Renal	12
uMM-01	Kappa	κII-A19	MM	1986	Lytic lesions and a metastic bone survey	Bone marrow	25
uMM-02	Kappa	κI-012/02	MM	2003	M spike and a negative bone x-ray	Bone marrow	25

AL, light chain amyloidosis; LCDD, light chain deposition disease; MM, multiple myeloma.

A day 100 post-stem cell transplant bone marrow sample was used in the cloning process for LCDD-02. We found cDNA sequences that matched two germline sequences, VκI O12/O2 and VκI L1. The sequence corresponding to the VκI O12/O2 germline was the most dominant (eight of 21 sequences) and was used as the reference sequence for this study (Figure 1). The nucleotide sequence comparisons for VκI L1 samples yielded all different sequences. It is interesting to note there are a difference of seven amino acids between the VκI O12/O2 and L1 germline sequences and in comparing their nucleotide sequences, there are 16 nucleotide changes. Germline nucleotide sequences were found in Vbase (www.mrccpe.cam.ac.uk). Since the bone marrow sample used to generate the LCDD-02 sequence was taken post-stem cell transplant, we think it was presumably polyclonal. Unfortunately, no pretreatment bone marrow sample was available for LCDD-02.

In Figure 1, the protein sequence for the variable domain of LCDD-02 is identical to MM-02 including the location and number of mutations in both of them. Both of these protein sequences correspond to the same germline, VκI O12/O2. However, when comparing the nucleotide sequences for MM-02 and LCDD-02, there are significant differences between them. We analyzed 12 LCDD-02 sequences and found nucleotide changes in different positions and at different frequencies confirming the polyclonal nature of the sample. Taking these differences into account, the sequences are different at a nucleotide level even though the amino acid sequences appear identical.

The constant domains for the AL and MM proteins were not sequenced. Most of the constant domain for MM-01 was determined by the Mayo Clinic Proteomics Research Center upon complete sequencing analysis with the kappa constant domain from the IMGT website (http://imgt.cines.fr/). However, based on the small region sequenced corresponding to the constant domain as part of the light chain variable domain (VL) sequencing, the constant domain of the lambda proteins was determined. There are six possible constant domains for lambda light chains. uAL-02 has a constant domain of IGLC1 and uAL-03 has a constant domain of IGLC2. For the MM proteins, the unique kappa constant domain was assigned. The sequences for all constant domains were found on the IMGT website (http://imgt.cines.fr/).

**Figure 2:**
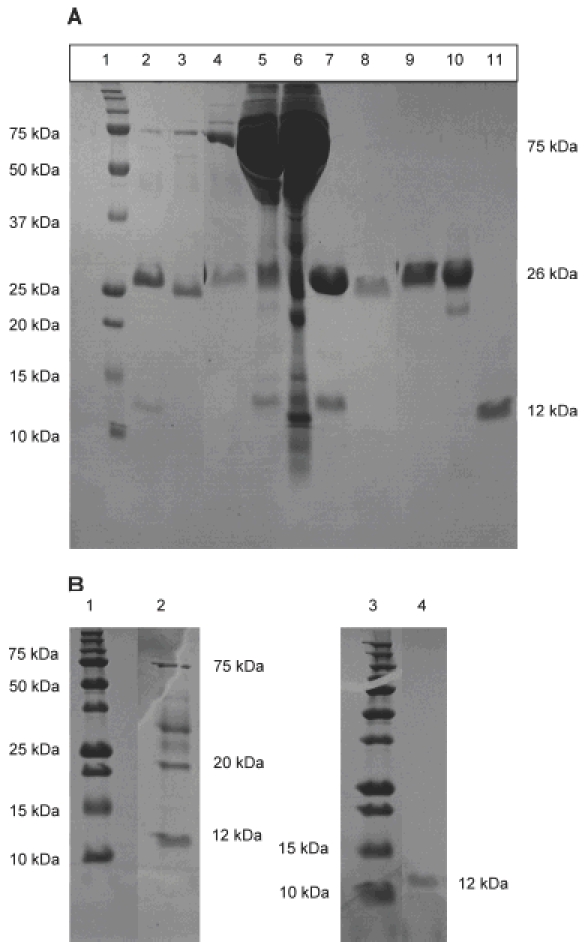
SDS-PAGE of urine and purified protein for each sample shows either a 26 kDa protein band for MM and AL proteins or a 12 kDa protein band for LCDD proteins. (A) Lanes 2–6, urine samples; lanes 7–11, purified protein samples. Lane 1, molecular weight marker; lanes 2 and 7, uMM-01; lanes 3 and 8, uMM-02; lanes 4 and 9, uAL-02; lanes 5 and 10, uAL-03; lanes 6 and 11, uLCDD-01. (B) uLCDD-02 gels: lanes 1 and 3, molecular weight marker; lane 2, urine sample; lane 4, purified protein.

The proteins were purified by size exclusion chromatography with 10 mM Tris–HCl pH 7.4 buffer. A 25–26 κDa protein band is visible in all samples except the purified LCDD proteins which has a 12 κDa protein band (Figure 2). A Western blot confirmed the purified proteins were immunoglobulin light chain proteins (data not shown). The molecular weight of the proteins was verified by analytical size exclusion chromatography (data not shown) with uAL-02 and uAL-03 being dimers, and uLCDD-01, uLCDD-02 and uMM-01 being monomers. uMM-02 migrated as a pentamer according to the analytical size exclusion column results, which could be contributed to the long-term storage of pure protein at 4°C before injecting onto the column (248 days).

Amino acid analysis results show 98% or more identity to the predicted sequence from translation of DNA for all proteins. Mass spectrometry of tryptic fragments had an overall good coverage of all proteins confirming the sequences from the DNA translation. The intact mass measurement of uAL-02, uAL-03 and uMM-01 were done by reverse-phase LC-electrospray-TOF mass spectrometry. The other three proteins did not give reliable results. The reduced samples of uAL-02, uAL-03 and uMM-01 had an observed mass close to the theoretical mass calculated for each of them.

**Figure 3:**
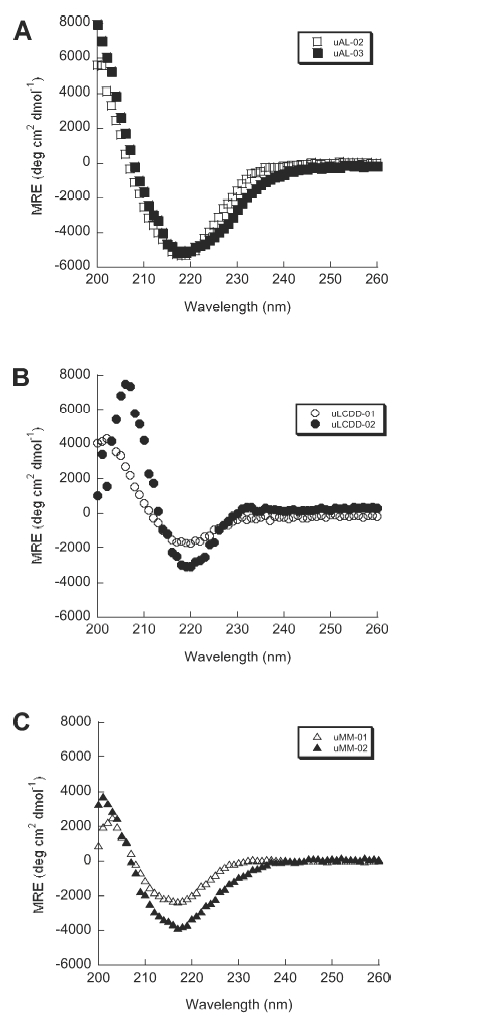
AL, LCDD and MM proteins have a β-sheet conformation by Far UV-CD. All of the BJP in this study display a β-sheet secondary structure based on the minimum ellipticity around 218 nm. (A) AL proteins, (B) LCDD proteins and (C) MM proteins. Experimental conditions: all Far UV-CD scans were performed at 4°C in a 0.2 cm path-length cuvette and in 10 mM Tris–HCl pH 7.4 buffer. Protein concentrations were determined to be between 5.5–25.6 μM.

**Figure 4:**
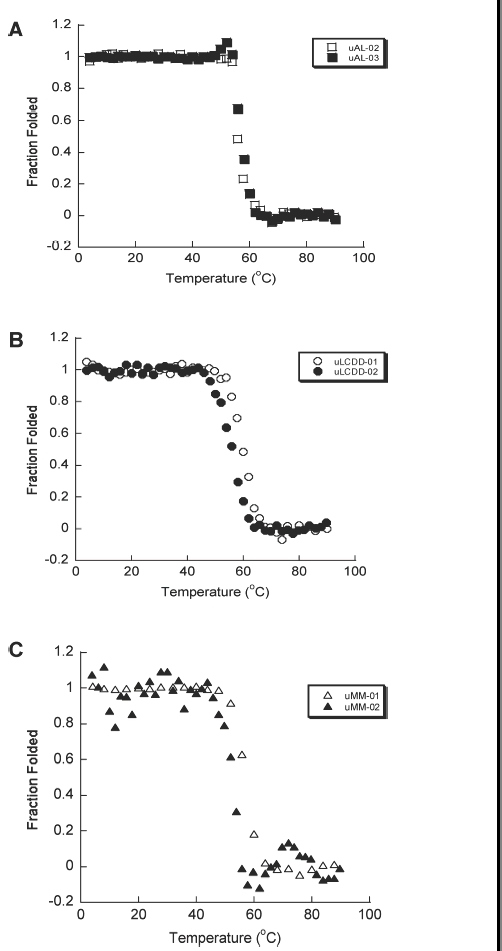
AL, LCDD and MM proteins have similar two state thermal unfolding transitions. Thermal denaturations were followed by CD following ellipticity at a wavelength of 218 nm. Unfolding and refolding curves were followed for each BJP. (A) AL proteins, (B) LCDD proteins and (C) MM proteins. All the BJP in this study were all able to refold reversibly except for uAL-03. The T_m_ for the proteins are 57.2°C for uAL-02, 57.3°C for uAL-03, 59.6°C for uLCDD-01, 56.0°C for uLCDD-02, 56.6°C for uMM-01 and 57.8°C for uMM-02. Protein concentrations are between 5.5 and 25.6 μM in 10 mM Tris–HCl pH 7.4 buffer in a 0.2 cm path-length cuvette.

The secondary structure of the six purified BJP was determined by Far UV-CD spectra (260–200 nm). We observed the expected β-sheet structure with a minimum around 218 nm for each protein (Figure 3). No notable differences were found in any of the spectra for these samples and all three groups of proteins look very similar. uLCDD-02 has a less intense mean residue ellipticity (MRE) value around 218 nm than the rest of the proteins and it has a shifted maximum around 206 nm. All proteins followed a two-state unfolding transition with similar T_m_ values (Figure 4 and Table II). All of the proteins were able to refold reversibly except for uAL-03. uLCDD-01 and uMM-01 have the lowest values/most favorable free energy of folding from the thermal denaturation data (ΔG(4°C)) while both AL proteins show the highest/less favorable values (Table II). The ΔG(H_2_O) calculated from chemical denaturation data follow the same trend but show different values. uAL-03 and uMM-02 show the highest C_m_ while uMM-01 and uMM-02 show the lowest ΔG_folding_ (Table II). Urea denaturations for uLCDD-01 and uLCDD-02 did not follow a two-state unfolding transition.

**Table II:** Thermodynamic parameters of Bence Jones proteins.

Protein	T_m_(°C)	ΔG(4°C) (kcal/mol)	C_m_(M)	ΔG(H_2_0) (kcal/mol)
uAL-02	57.2 ± 0.5	−6.8	3.1 ± 0.5	−3.1
uAL-03	57.3 ± 0.4	−6.6	4.4 ± 0.5	−2.2
uLCDD-01	59.6 ± 0.4	−17.4	NA*	NA*
uLCDD-02	56.0 ± 1.4	−12.6	NA*	NA*
uMM-01	56.6 ± 0.4	−17.1	4.0 ± 0.8	−3.6
uMM-02	57.8 ± 0.6	−15.1	6.2 ± 0.5	−4.0

* Unable to determine these values due to lack of two-state transition. AL, light chain amyloidosis; LCDD, light chain deposition disease; MM, multiple myeloma.

**Figure 5:**
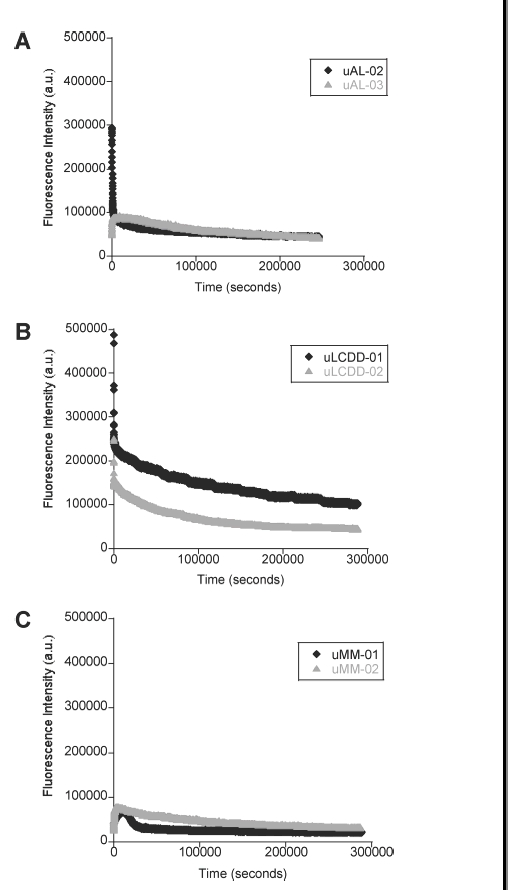
Aggregate formation followed by tryptophan fluorescence. (A) AL proteins, (B) LCDD proteins and (C) MM proteins. Experimental conditions: 10 mM Tris–HCl pH 7.4 buffer, 15 μM ThT, 5 μM protein; incubated at T_m_ (57°C for uAL-02, uAL-03, uMM-01 and uMM-02, 60°C for uLCDD-01 and uLCDD-02); stirring at 300 rpm for 72 h with a reading occurring every 25 s with an integration time of 0.5 s; excitation wavelength 294 nm and emission wavelength 350 nm.

We conducted aggregation assays by incubating each protein (5 μM) with 15 μM ThT in 10 mM Tris– HCl pH 7.4 at their T_m_ (578C for uAL-02, uAL-03, uMM-01, uMM-02, 60°C for uLCDD-01, uLCDD-02) (Table II) as was previously reported [13,14]. The kinetics of aggregate formation were followed simultaneously by tryptophan and thioflavine T (ThT) emission wavelengths of 350 nm and 480 nm, respectively. The changes on tryptophan fluorescence for the two AL proteins studied have different early aggregation events but eventually converge into the same process (Figure 5, panel A). The changes in tryptophan fluorescence followed the same trend for the two LCDD proteins but LCDD-01 had higher fluorescence intensity throughout the whole assay when compared to LCDD-02 (Figure 5, panel B). MM proteins had a slight variation in the beginning of the aggregation process but the fluorescence signals eventually converge (Figure 5, panel C). The initial changes seen in Figure 4, panel A and B (AL and LCDD graphs) are due to the initial equilibration of the protein samples to their T_m_. The most significant changes in the BJP happened within the first 2 h of the reaction. In conclusion, no pattern of tryptophan fluorescence can be matched to the different aggregation processes by the different BJP proteins.

**Figure 6:**
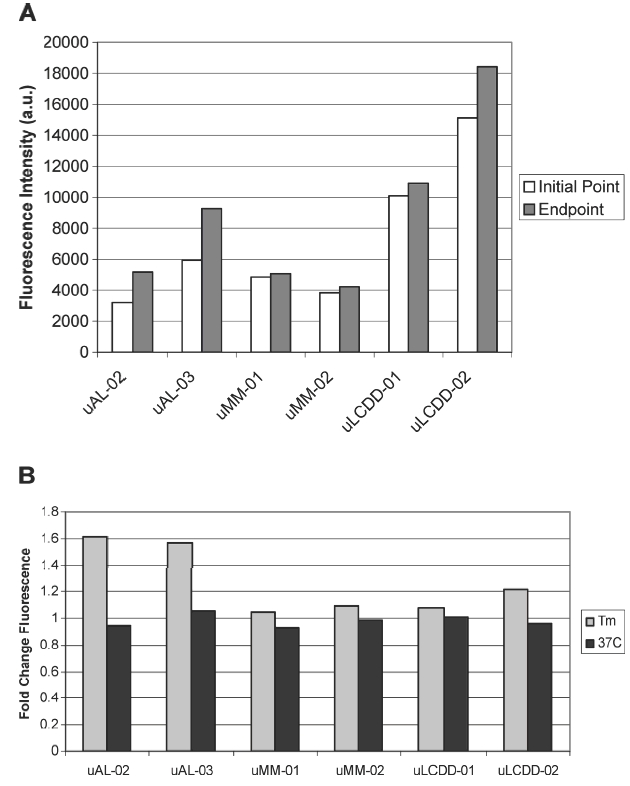
Aggregate formation followed by thioflavine T fluorescence (ThT). (A) Initial and end-point ThT fluorescence readings at their T_m_. (B) Fold change between initial and endpoint ThT fluorescence readings from samples incubated at either their T_m_ or at 37°C. Experimental conditions: 10 mM Tris–HCl pH 7.4 buffer, 15 μM ThT, 5 μM protein; incubated at T_m_ (57°C for uAL-02, uAL-03, uMM-01 and uMM-02, 60°C for uLCDD-01 and uLCDD-02) with stirring at 300 rpm for 72 h with a reading occurring every 25 s with an integration time of 0.5 s.

**Figure 7:**
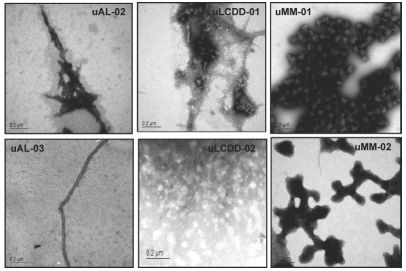
AL proteins show amyloid fibrils. LCDD proteins show amorphous aggregates and MM proteins show spherical species. uAL-03 and uLCDD-02 were stained with 4% uranyl acetate. uAL-02, uLCDD-01, uMM-01 and uMM-02 were stained with 1% phosphotungstic acid. The negatively stained grids were examined using a JEOL 1200 EX transmission electron microscope. Scale bars are located in the lower left corner of each image.

The intensity of the initial and endpoint ThT fluorescence for each BJP is summarized in Figure 6 along with the fold change for each protein. The largest fold change is found in both of the AL proteins. The formation of fibrils or aggregates was confirmed by EM as seen in Figure 7. Both of the AL proteins formed fibrils with a diameter ranging from 14–50 nm. LCDD proteins formed amorphous aggregates with diameters between 50–300 nm. MM proteins formed spherical species with diameters of 100–150 nm. MM and LCDD proteins are considered non-pathologic due to the lack of the formation of fibrils.

There is no significant increase in ThT fluorescence after 8 days following the aggregation kinetics of the samples at 37°C. The fold change for the proteins at 37°C is lower than the fold change at their T_m_. This would suggest the ability to form aggregates under these conditions is occurring slower than samples incubated at their T_m_ with continuous agitation of 300 rpm (Figure 6).

## Discussion

The predicted amino acid sequences from the cloned MM-02 and LCDD-02 samples are identical for the variable domain. However, it is important to note a few differences between these two samples. The nucleotide sequences are different between these two samples. The MM-02 protein also contains the constant domain which is missing in the LCDD-02 protein. It is possible that the dominant clone found for LCDD-02 is not the pathologic clone but it may be the most abundant sequence in the polyclonal bone marrow specimen taken post-stem cell transplant. MM-02 is a clonal sequence in that almost all of the nucleotide sequences are identical. This is not true for the LCDD-02 nucleotide sequences.

Our results indicate that BJP derived from patients afflicted with these three different light chain diseases present very similar T_m_ values. Both thermal and chemical denaturation derived ΔG_folding_ values indicated that MM and LCDD proteins are more stable than AL proteins, which is in agreement with previous reports [12,15–17].

Due to the absence of two-state unfolding transition for LCDD proteins using urea denaturation, we wanted to know if they were sampling unfolded states and exposing hydrophobic patches in the absence of denaturant at room temperature. For that purpose, the proteins were incubated with 2.3 μM ANS (8-anilino-1-naphthalene-sulfonic acid) in the presence and absence of 6 M urea to see if enhanced fluorescence with ANS could be detected. ANS is a hydrophobic dye used to detect exposed hydrophobic surfaces as well as partially folded intermediates [18–20]. Fluorescence enhancement of ANS in the presence of both proteins suggested that LCDD proteins are sampling partially unfolded states (data not shown). The presence of 6 M urea further enhanced ANS fluorescence, which suggested that the proteins were able to expose more hydrophobic surfaces and therefore might be partially unfolded in the absence of denaturant (data not shown). Since a two-state unfolding transition was observed when a thermal denaturation was performed, it is possible that the unfolded state populated in urea is different from the one populated at high temperatures. The LCDD proteins in this study lack the constant domain, which may help explain their lack of a two-state unfolding transition when followed by urea.

Striking differences between the different BJP were found in their aggregation properties. The different BJP were incubated at their T_m_ in 10 mM Tris–HCl pH 7.4 buffer for 72 h. These solution conditions have been reported to maximize aggregation [13,14,21]. Our results show the largest fold change in the ThT fluorescence intensity between the initial and endpoints for the AL proteins.

According to our results, the tryptophan emission fluorescence decreases in intensity over time during the *in vitro* aggregation assay for all the BJP in this study. The changes in the signal can be in response to conformational transitions, denaturation or changes in the environment of the protein [22].

The proteins studied were modeled using 1BRE.pdb (κ) and 1CD0.pdb (λ) to determine the location of each tryptophan residue in the protein to get an idea of which ones were contributing to the fluorescence which may help us understand the conformational changes occurring during aggregation. The six proteins studied have a tryptophan residue at position 35. Both of the AL proteins have four tryptophan residues which are in the same position in each of these sequences even though they belong to two different variable and constant domain germline sequences. The two tryptophan residues in the MM proteins are in the same position even though they belong to different germline sequences. LCDD-01 has two tryptophan residues one at position 35 protein and tryptophan 50. LCDD-02 only has one tryptophan in the variable region at position 35.

AL proteins formed amyloid fibrils with diameters of 14–50 nm. These diameters are slightly larger than the range of 7–12 nm that has been previously reported for the variable domain amyloid fibrils [23]. The difference in diameter could be due to the presence of the constant domain as part of the fibril. MM proteins consistently formed large spherical species with diameters of 100–150 nm, which is contrary to previous reports of variable domain MM proteins that were able to form amyloid fibrils [16,24–26]. LCDD proteins formed predominantly amorphous aggregates that had diameters of 50– 300 nm. MM and LCDD proteins did not form fibrils and in turn are non-pathogenic.

**Figure 8:**
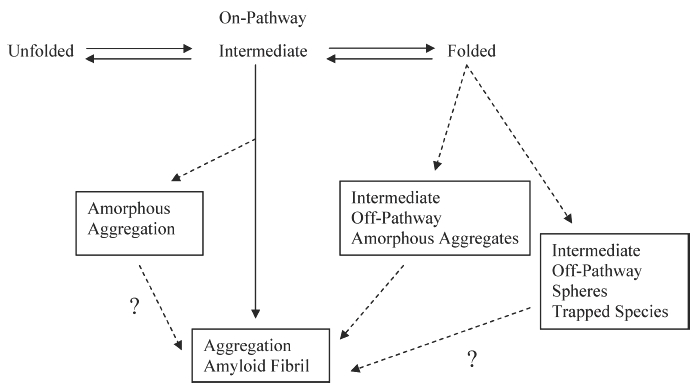
Model for protein folding and misfolding where aggregation may occur through an on or off pathway intermediate.

What causes a protein to misfold and form either fibrils or aggregates? Clues to the type of aggregation formed by a protein could be found in the pathway the protein follows in becoming an aggregate. The aggregation pathway could be either an on- or off-folding pathway, going through a possible intermediate before reaching its form of deposition. Khurana et al. has reported that partially unfolded intermediates could lead to fibrils or amorphous aggregates [12]. According to Vidal et al., off-pathway aggregation does not necessarily form amyloid fibrils. For example, in LCDD, the formation of fibrils may be avoided due to the formation of intermediates that lead to aggregate formation in an off-pathway fashion [27]. Figure 8 shows a model of the various misfolding pathways a protein could follow on its way to becoming an aggregate. It is possible the spheres formed by MM proteins and LCDD amorphous aggregates are a trapped intermediate state (most likely off-pathway) after 72 h of incubation and are unable to proceed to fibrils. It is not known if over time the protein will be able to leave this trapped state and go on to form fibrils.

The mutations for the various BJP in this study can be located throughout the protein, but they tend to cluster in certain areas of the variable domain [28].

The variable domain has an immunoglobulin fold consisting of two antiparallel β-sheets packed together and joined by a disulfide bond [29]. AL proteins have the largest number of mutations out of the six proteins studied and most of their mutations are located in the β-sheet that is part of the heavy chain/light chain dimer interface. uLCDD-01 has mutations in the top or bottom of the beta barrel. uMM-01, uMM-02, and uLCDD-02 have their mutations located in the N and C terminus strands.

In conclusion, we have determined thermodynamic parameters for human derived BJP, we have characterized the aggregation properties and described their differences. This study has shed some light into the differences among BJP involved in different light chain diseases.

## Abbreviations

AL: light chain amyloidosis
BJP: Bence Jones proteins
CD: circular dichroism
CDR: complementarity determining regions
C_m_: melting concentration of denaturant
EM: electron microscopy
FF: fraction folded
FR: framework regions
LCDD: light chain deposition disease
MM: multiple myeloma
RTPCR: reverse transcriptase polymerase chain reaction
T_m_: melting temperature
ThT: Thioflavine T
Ve/Vo: elution to void volume ratio
